# Atovaquone enhances antitumor efficacy of TCR-T therapy by augmentation of ROS-induced ferroptosis in hepatocellular carcinoma

**DOI:** 10.1007/s00262-024-03628-2

**Published:** 2024-02-13

**Authors:** Anan Chen, Zhiwu Yu, Na Ma, Xinyu Lu, Yajing Zhang, Weikang Xu, Yiyue Wang, Jiayi Xie, Yuqi Qin, Guoheng Mo, Sha Wu, Jinlin Hou, Wei Zhu

**Affiliations:** 1 State Key Laboratory of Organ Failure Research, Guangdong Provincial Key Laboratory of Viral Hepatitis Research, Department of Infectious Diseases, Nanfang Hospital, Southern Medical University, Guangzhou, 510515 China; 2 Department of Laboratory Medicine, Affiliated Cancer Hospital and Institute of Guangzhou Medical University, Guangzhou, 510095 Guangdong China; 3 Department of Pathology, The First People’s Hospital of Foshan, Foshan, 528000 China; 4 State Key Laboratory of Oncology in Southern China, Collaborative Innovation Center for Cancer Medicine, Sun Yat-Sen University Cancer Center, Guangzhou, 510060 China; 5 Department of Gastroenterology, The Second Affiliated Hospital of Guangzhou Medical University, Guangzhou, 510220 China; 6 Department of Immunology, School of Basic Medical Sciences, Guangdong Provincial Key Laboratory of Proteomics, Southern Medical University, Guangzhou, 510515 China

**Keywords:** Hepatocellular carcinoma (HCC), T-cell receptor (TCR)-engineered T-cells (TCR-T), Atovaquone, Ferroptosis, RNA-seq, Combination therapy

## Abstract

T-cell receptor (TCR) engineered T-cell therapy has recently emerged as a promising adoptive immunotherapy approach for tumor treatment, yet hindered by tumor immune evasion resulting in poor therapeutic efficacy. The introduction of ferroptosis-targeted inducers offers a potential solution, as they empower T cells to induce ferroptosis and exert influence over the tumor microenvironment. Atovaquone (ATO) stands as a prospective pharmaceutical candidate with the potential to target ferroptosis, effectively provoking an excessive generation and accumulation of reactive oxygen species (ROS). In this study, we evaluated the effectiveness of a combination therapy comprising ATO and TCR-T cells against hepatocellular carcinoma (HCC), both in vitro and in vivo. The results of lactate dehydrogenase and cytokine assays demonstrated that ATO enhanced cytotoxicity mediated by AFP-specific TCR-T cells and promoted the release of IFN-*γ* in vitro. Additionally, in an established HCC xenograft mouse model, the combined therapy with low-dose ATO and TCR-T cells exhibited heightened efficacy in suppressing tumor growth, with no apparent adverse effects, comparable to the results achieved through monotherapy. The RNA-seq data unveiled a significant activation of the ferroptosis-related pathway in the combination therapy group in comparison to the TCR-T cells group. Mechanistically, the synergy between ATO and TCR-T cells augmented the release of IFN-*γ* by TCR-T cells, while concurrently elevating the intracellular and mitochondrial levels of ROS, expanding the labile iron pool, and impairing the integrity of the mitochondrial membrane in HepG2 cells. This multifaceted interaction culminated in the potentiation of ferroptosis within the tumor, primarily induced by an excess of ROS. In summary, the co-administration of ATO and TCR-T cells in HCC exhibited heightened vulnerability to ferroptosis. This heightened susceptibility led to the inhibition of tumor growth and the stimulation of an anti-tumor immune response. These findings suggest that repurposing atovaquone for adoptive cell therapy combination therapy holds the potential to enhance treatment outcomes in HCC.

**Supplementary Information:**

The online version contains supplementary material available at 10.1007/s00262-024-03628-2.

## Introduction

Primary liver cancer ranks as the sixth most frequently diagnosed cancer and stands as the third leading cause of cancer-related mortality worldwide, as indicated by the most recent data in the global cancer statistics [1], mainly including hepatocellular carcinoma (HCC) (accounting for 75–85% of cases) and intrahepatic cholangiocarcinoma (10–15%) [2]. In China, HCC accounts for 45.3% of the global incidence [3]. Surgical resection and ablation represent the primary treatment options for patients diagnosed with early-stage hepatocellular carcinoma, However, a significant portion of HCC patients receive their diagnosis at an advanced stage, missing the optimal window for surgical intervention [4]. Furthermore, the elevated recurrence rate further diminishes the benefits of surgical treatment [4]. With a paucity of therapeutic options, patients facing advanced-stage HCC confront a grim prognosis. Consequently, there exists an urgent imperative for the development of innovative and efficacious treatment modalities to enhance the prognosis of advanced-stage HCC patients.

Immunotherapy, including immune-checkpoint inhibitors (ICIs) and adoptive cell therapy (ACT), has revolutionized the clinical management of various tumors and offers novel prospects for HCC treatment [5, 6]. However, the application of ICIs in HCC therapy presents challenges, as the majority of patients exhibit limited responsiveness to ICIs, either as monotherapy or in combination regimens, owing to a low response rate and the occurrence of severe immune-related adverse events [7, 8]. Even with combination therapy, the highest attainable objective response rate remains below 40% [9]. As for ACT, encompassing the utilization of transgenic tumor antigen specific TCR-T or CAR-T, it is noteworthy that, to date, clinical benefits have exclusively been observed in advanced HCC patients through the application of CAR-T cells engineered to target Glypican-3 (GPC3) [10].

TCR-T cell therapy has emerged as a more formidable strategy in treating solid tumor [11]. Currently, numerous preclinical and clinical trials are underway, including several phase I/II clinical trials targeting HBV antigens and alpha-fetoprotein (AFP) are now in progress to evaluate the adoptive transfer of T cells with a modified TCR (NCT03634683, NCT02719782, NCI03132792, NCT03971747, NCT03899415) [12]. AFP is believed to be an attractive target for HCC therapy, given its high expression in HCC patients [13]. Nevertheless, therapeutic interventions based on TCR-T for AFP/HLA have been effective in only a small fraction of treated patients [14]. As a result, there remains a pressing need to devise strategies that can enhance the clinical advantages of TCR-T therapy for patients grappling with advanced hepatocellular carcinoma [15].

Atovaquone is an FDA-approved anti-malarial drug with its target on cytochrome bc1 complex [16]. Some studies have proposed that ATO exhibits potent anti-tumor properties in various types of cancer cells, including thyroid, hematological, colorectal, lung, breast, bladder, pancreatic, and HCC [17–21]. Mechanistically, ATO has been shown to inhibit the mitochondrial electron transport chain at mitochondrial complex III [22] which is the major site for ROS generation at the electron transport chain [23]. Consequently, ATO has recently undergone repurposing as an anti-tumor pharmaceutical agent. Due to its reported ability to increase oxidative stress in vitro [24] and reduce hypoxia in vivo [19], in addition to its excellent safety profile [16], thus, we hypothesize that ATO might improve TCR-T cells anti-tumor response. It is of considerable significance to delve into the potential role of ATO in TCR-T therapy for HCC patients and to investigate a combined treatment approach that leverages ATO in conjunction with TCR-T cells, with the aspiration of achieving an even more efficacious curative effect.

Ferroptosis, a form of programmed necrosis, primarily ensues due to extra-mitochondrial lipid peroxidation triggered by iron-dependent ROS accumulation [25]. The build-up of cellular ROS plays a pivotal role in eliciting lipid peroxidation (LPO), with effective accumulation and reduced clearance of LPO being essential for the initiation of ferroptosis [26]. Recent findings further underscore that mitochondrion-mediated ROS production is indispensable for lipid peroxidation and ferroptosis induction and the ultimate orchestration of ferroptotic cell death [27, 28]. Elevated levels of reactive oxygen within mitochondria constitute a significant mechanism employed by certain therapeutic agents, such as sorafenib treatment in HCC, to render tumor cells susceptible to ferroptosis [29]. Elicitation of ferroptosis by IFN-*γ* secreted from infiltrating CD8^+^ T-cells has also been shown to enhance the anti-tumor activity of checkpoint blockade and radiotherapy [30, 31]. These investigations underscore the clinical significance of ferroptosis in impeding HCC progression and augmenting the effectiveness of immunotherapy [32].

In this study, we conducted an evaluation of the effects of ATO on human TCR-T cells. Both in vitro and in vivo experiments consistently revealed that the combination of ATO and TCR-T cells yielded an augmented anti-tumor response compared to monotherapy while showing minimal observable side effects. RNA-seq results suggested that ferroptosis emerged as the main pattern of tumor death under ATO + TCR-T treatment for HCC. At the cellular level, our data showed that ATO upregulates cancer cells HLA-I expression and increases TCR cells IFN-*γ* production, iron and ROS accumulation, and loss of mitochondrial membrane potential (ΔΨm) during TCR-T killing. We propose a sequential model in which (i) ATO induces ROS accumulation within tumor cells and upregulates HLA-I expression, (ii) subsequently promotes TCR-T recognition and IFN-γ release, (iii) ultimately triggers and reinforces IFN-*γ*-mediated ferroptotic cell death. These data provide substantial evidence supporting the potential clinical utility of TCR-T cells in combination with low‐dose ATO as HCC therapy.

## Materials and methods

### Cell and mice

The HCC cell line LM3 was purchased from the China Center for Type Culture Collection (Wuhan, China). HepG2 was obtained from the Shanghai Cell Bank of the Academy of Chinese Sciences and Liver Cancer Institute, Zhongshan Hospital, Fudan University (Zhongshan, China). Both cells were cultured in Dulbecco’s modified Eagle medium (Corning) supplemented with 10% fetal bovine serum (FBS) (ExCell Bio). All cell lines were cultured in a humidified atmosphere of 5% CO_2_ at 37 °C. All experiments were performed with mycoplasma-free cells.

B-NDG mice (NOD.CB17-PrkdcscidIl2rgtm1/Bcgen; 7 weeks old; female) were purchased from Jiangsu Biocytogen Laboratory Animal Co. and maintained under specific pathogen–free conditions in accordance with the animal experimental guidelines of Southern Medical University. All animal procedures were approved by the Institutional Animal Care and Use Committee (IACUC).

### Generation of AFP-specific TCR-T cells

Production of AFP-specific TCR-T cells is similar to that described previously [33]. In brief, peripheral blood mononuclear cells (PBMCs) were isolated from healthy donors by density gradient centrifugation with Ficoll-hypaque. T cells were purified with T cell isolation kit (STEMCELL Technologies, Cambridge, MA) and stimulated with CD3/CD28 tetrameric antibody complex (STEMCELL Technologies, Cambridge, MA) for 24 h. Activated T cells were transduced with lentiviral particles containing the TCR gene. After 48 h, the expression of TCR was measured by flow cytometry and CD3/CD28 antibody complex was rinsed away. For TCR-T cells culture, RPMI-1640 medium containing 10% FBS and 50 IU/mL of interleukin-2 (IL-2) (Quanqi, Shandong, China) was refreshed every other day.

### Apoptosis assay

The apoptosis assay was carried out using the Annexin V-FITC (BioLegend, 640945) and 7- Aminoactinomycin D (7-AAD) (BioLegend, 420404) according to the manufacturer’s instructions. After cocultured with ATO (MCE, HY-13832), TCR-T cells, or ATO plus TCR-T cells for 12 h, HepG2 cells were collected, and incubated with Annexin-V and 7-AAD in the dark for 15 min. Cells were detected on a FACSCantoTM II FlowCytometer (BD, United States).

### Proliferation assay

HepG2 cells were stained with 2.5 μM CFSE (Invitrogen, C34554) at 37 °C for 5 min, and then washed twice with fresh medium. The labeled cells were cultured in the presence of various concentrations of ATO (0, 2.5, 5, 10 µM) for 96 h, and then collected to analyze proliferation using FACSCantoII Flow cytometry (BD, United States).

### Flow cytometry analysis

For membrane staining, TCR-T cells were stained with anti-CD8 (SK1 or HIT8a), anti-TCR βchain (H57-597), anti-CD45RA (HI100), anti-CCR7 (G043H7), PD-1 (NAT105), anti-Tim-3(F38-2E2), anti-LAG-3(11C3C65), and anti-CTLA-4 (BNI3), as well as tumor cells were stained with anti-FsaL (NOK-1), anti-HLA-A02 (BB7.2), and anti-HLA-A24 (17A10). For intracellular staining, TCR-T cells were cocultured with HepG2 in the presence or absence of ATO for 6 h. The stimulated T cells underwent membrane staining first, and then fixed and permeabilizated, followed by staining with anti-Perforin (B-D48), anti-IL-2(MQ1-17H12), anti-granzyme B (GB11), anti-IFN-*γ* (4S.B3), and anti-TNF-*α* (MAb11). For the detection of cleaved caspase-3, the treated HepG2 cells were fixed and permeabilizated, followed by staining with cleaved caspase-3 (Asp175) antibody and then with Alexa Fluor 594-conjugated secondary antibody. Flow cytometry analysis was performed using FACSCantoII Flow cytometry (BD, United States) and analyzed using FlowJo (Ashland, United States). All antibodies were purchased from BioLegend.

### Elisa analysis

TCR-T cells and HepG2 cells were co-incubated at a ratio of 1∶1 in presence of various concentrations of ATO (0, 2.5, 5, 10 µM) for 24 h. Supernatants were harvested for detection of cytokine release. The levels of IL-2, IFN-*γ* and TNF-*α* were respectively determined using IL-2 ELISA kit (431803, BioLegend), IFN-*γ* ELISA kit (430101, BioLegend), and TNF-*α* ELISA kit (430201, BioLegend) according to the manufacturer’s instructions. Absorbance at 450 nm was measured (BioTek, Vermont, United States). All experiments were performed in triplicate.

### Cytotoxicity assay

Cytotoxicity was determined using CytoTox 96 Non-Radioactive Cytotoxicity Assay kit (Promega, G1780, United States) as described previously [34]. In brief, TCR-T cells and HepG2 cells were cocultured at various ratios in presence of various concentrations of ATO (0, 2.5, 5, 10 µM) for 24 h and collected the supernatants for detection of lactate dehydrogenase release according to manufacturer’s protocol. Ferrostatin-1 (2 μM, MCE, HY-100579), liproxstatin-1 (2 μM, MCE, HY-12726), deferoxamine mesylate (10 μm, MCE, HY-B0988), or UAMC-3203 (1 μM, MCE, HY-112909) were added in ferroptosis rescue experiments. The percentage of specific killing was calculated using the following formula: [(experimental release − effector spontaneous release − target spontaneous release)/(target maximum release − target spontaneous release)] × 100%.

### *In vivo* treatment of ATO and TCR-T cells

HepG2 cells (5 × 10^6^ cells) were inoculated subcutaneously in the right flank area of B-NDG mice. When average tumor volume had reached about 90 mm^3^, the mice were randomly divided into 4 groups (day 0). Each group was treated with vehicle, ATO, TCR-T cells, or ATO plus TCR-T cells. ATO was oral administered in the peanut butter at 50 mg/kg every day for 7 successive days. CD8^+^ TCR-T cells were injected intravenously with 4 × 10^6^/200 μL on day 0. Tumor size and body weight were measured every 2 days.

### Immunohistochemistry stain

The fixed and embedded tumors were sectioned into 4 μm sections. Tumor sections were quenched by peroxidase, and then blocked for 1 h in goat serum (Boster, AR0009). The tumor sections were incubated with anti-CD3(Abcam, ab16669, 1∶100) or anti-Ki67 (Proteintech, 27309–1-AP, 1∶1000) and biotinylated secondary antibody at 37 °C, 1 h for each incubation. Tumor sections were further stained with streptavidin-HRP and diaminobenzidine (DAB) peroxidase, followed by counterstaining with hematoxylin for cell nuclei. All sections were examined microscopically.

### Cell-line RNA-seq

Total RNA was extracted from cell line by the Trizol isolation reagent (Invitrogen, 15596026) based on the manufacturer’s instructions. Subsequent RNA sample processing, RNA sequencing, and transcriptome data analysis services were all provided by Gene Denovo Biotechnology Co. (Guangzhou, China).

### Bioinformatic analysis

Bioinformatic analysis was performed using Omicsmart, a real-time interactive online platform for data analysis (http://www.omicsmart.com). Gene Set Enrichment Analysis (GSEA), Gene Ontology (GO) and Kyoto Encyclopedia of Genes and Genomes (KEGG) enrichment analysis from this platform was carried out to determine the distributions of unigenes based on their functions and biological pathways.

### Reactive oxygen species (ROS) measurements

Intracellular formed ROS can be measured using fluorescent dyes, including DCFH-DA (Beyotime, S0033S) and MitoSOX (Abclonal, RM02822). In brief, HepG2 or LM3 cells were cocultured with ATO, TCR-T cells, or ATO plus TCR-T cells in 48-well plates for 6 h, and washed once with PBS. Then subsequently incubated with fresh medium containing 10 μM DCFH-DA and 5 μM MitoSOX at 37 °C for 20 min. After washing, cells were imaged using fluorescence microscopy and analyzed by flow cytometry.

### Mitochondrial transmembrane potential (Δψm) assay

TMRE indicator (Beyotime, C2001S, China) was used to detect mitochondrial transmembrane potential (Δψm) according to the manufacturer’s protocol. After indicated treatments, HepG2 cells were washed once with PBS and then incubated with TMRE working solution (10 μM TMRE indicator in fresh medium) at 37 °C for 30 min, and fluorescence intensity was determined with a flow cytometer. Quantification for the ΔΨm assay was carried out to measure the fluorescence intensity ratio of related to vehicle group.

### Intracellular labile iron pool (LIP) detection

Intracellular LIP was measured using Calcein-AM (Biosharp, BL130A) and 7- AAD (BioLegend, 420404). In brief, HepG2 cells were cocultured with ATO, TCR-T cells, or ATO plus TCR-T cells in 48-well plates for 12 h, and washed once with PBS. Then subsequently incubated with fresh medium containing 0.5 µM Calcein-AM and 1 µM 7-AAD at 37 °C for 10 min. Cells were analyzed by flow cytometry. Green fluorescence correlates with impaired Calcein-AM due to reaction of calcein with labile iron. Red fluorescence from 7-AAD represents dead cells.

### Statistical analysis

Statistical analysis was performed using GraphPad Prism 8.0 (GraphPad Software, La Jolla, CA, USA) with Student’s *t* test or two-way ANOVA. Error bars presented the SD of three biological replicates. *p* values < 0.05 was considered significant; *p < 0.05; **p < 0.01; ***p < 0.001.

## Results

### Atovaquone (ATO) promotes AFP TCR-T function in vitro

**Fig. 1:**
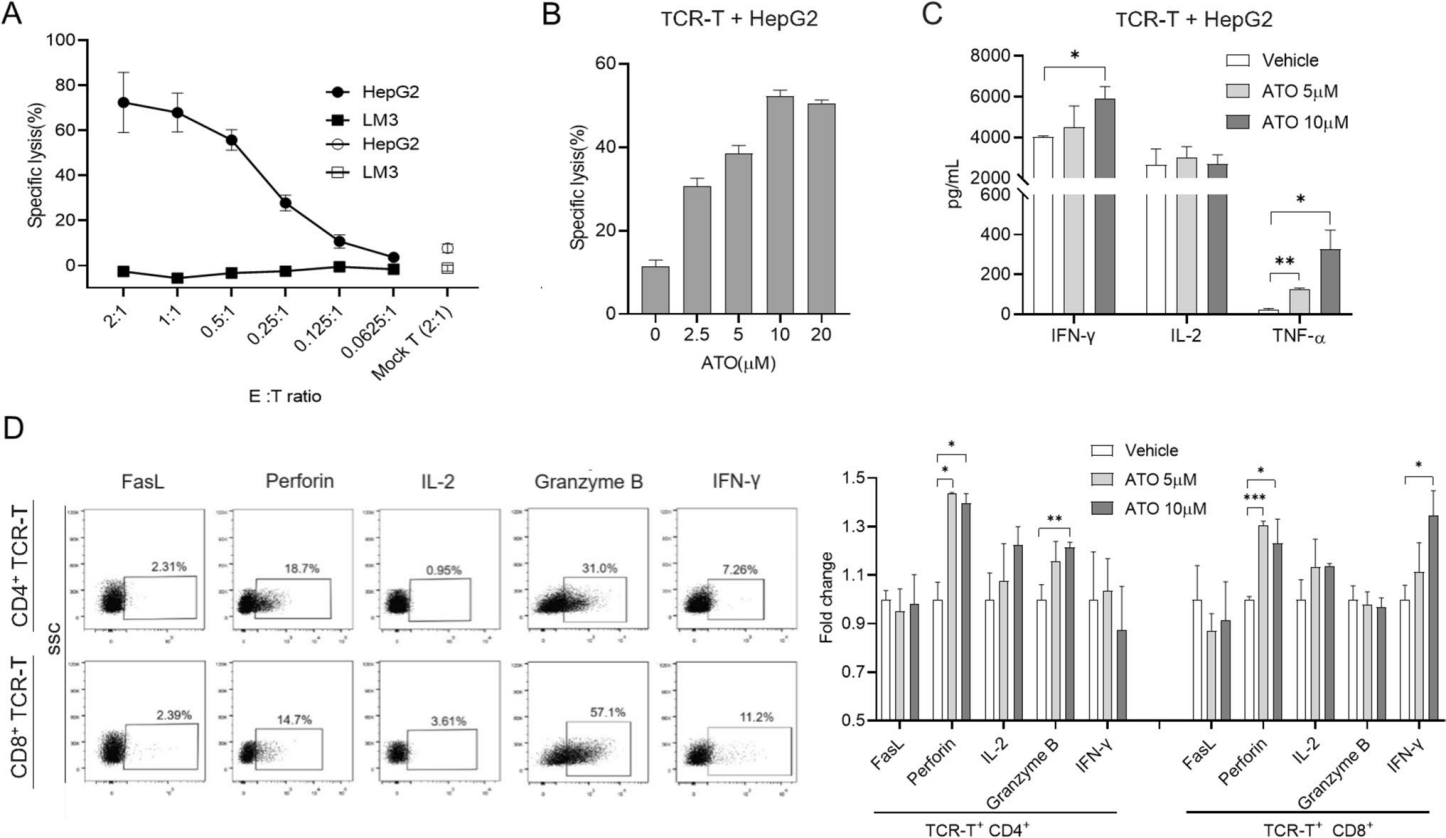
Effects of ATO in TCR-T cells function in vitro. **A** AFP TCR-T cells lysed cancer cell lines in a manner dependent on the E∶T ratio. Lysis was assessed 24 h after TCR-T cells inoculation using the LDH release assay. HepG2, HLA-A02^+^/AFP^+^; LM3, HLA-A02^−^/AFP^+^. **B** HepG2 cells were treated with AFP TCR-T cells (E∶T = 0.5∶1) in the presence of ATO with different concentrations for 24 h, and then analyzed by LDH release assay. **C** The supernatant from (**B**) was tested by ELISA for the amount of IL-2, TNF-*α*, and IFN-*γ*. **D** TCR-T cells were co-cultured with HepG2 in the presence of different doses of ATO or vehicle. FasL, perforin, granzyme B, IL-2, and IFN-*γ* expression were analyzed by flow cytometry and each group was analyzed by positivity rate normalized to the vehicle group. Each experiment was performed in triplicate. The experiment was repeated twice with similar data. Data are presented as mean ± SD. * for *p* < 0.05, ** for *p* < 0.01 and *** for *p* < 0.001

To determine whether ATO affects the function of TCR-T therapy, we constructed AFP-specific TCR-T cells (Fig. S1A) by transducing a murine TCR [33] into human primary T cells. Consistent with the previously described findings [33], this TCR-T cells could specifically kill HLA-A*0201^+^/AFP^+^ HepG2 in a dose-dependent manner (Fig. 1A). Then, the TCR-T cells and HepG2 were treated with ATO, respectively. ATO could not induce significant cytotoxic activity of HepG2 at a high concentration of 10 µM, but promoted apoptosis and hindered proliferation of HepG2 cells (Fig. S1B, C and D), a trend in accordance with the observations made by Gao et al. [21]. In addition, there was no significant changes in the short-term apoptosis and long-term cell activity of TCR-T cells in the presence of 10 µM ATO (Fig. S1E, F). To assess the anti-tumor effects of the ATO and TCR-T combination, the TCR-T cells were co-cultured with HepG2 cells in the presence of varying ATO concentrations. As anticipated, ATO significantly enhanced the cytotoxicity of TCR-T cells in a dose-dependent manner (Fig. 1B). However, when we examined the apoptosis marker cleaved caspase-3, we found that it was not increased in the group that receiving ATO + TCR-T cells (Fig. S1G, H), suggesting that ATO in combination with TCR-T promotes a non-apoptotic mode of cell death. Furthermore, TCR-T cells also produced higher levels of IFN-*γ* and TNF-α in the presence of ATO (Fig. 1C). To gauge the intrinsic cytotoxic capabilities of TCR-T cells, we examined the expression of corresponding effector molecules, including FasL, perforin, granzyme B, IL-2, and IFN-*γ* (Fig. 1D). CD4^+^ TCR-T cells produced more perforin and granzyme B when exposed to ATO, whereas CD8^+^ TCR-T showed increased expression of perforin and IFN-*γ*. These results demonstrate that ATO has the potential to enhance the cytotoxic activities of TCR-T cells against HCC by promoting a non-apoptotic mode of cell death.

### The effect of ATO on the distribution of TCR-T subsets

**Fig. 2:**
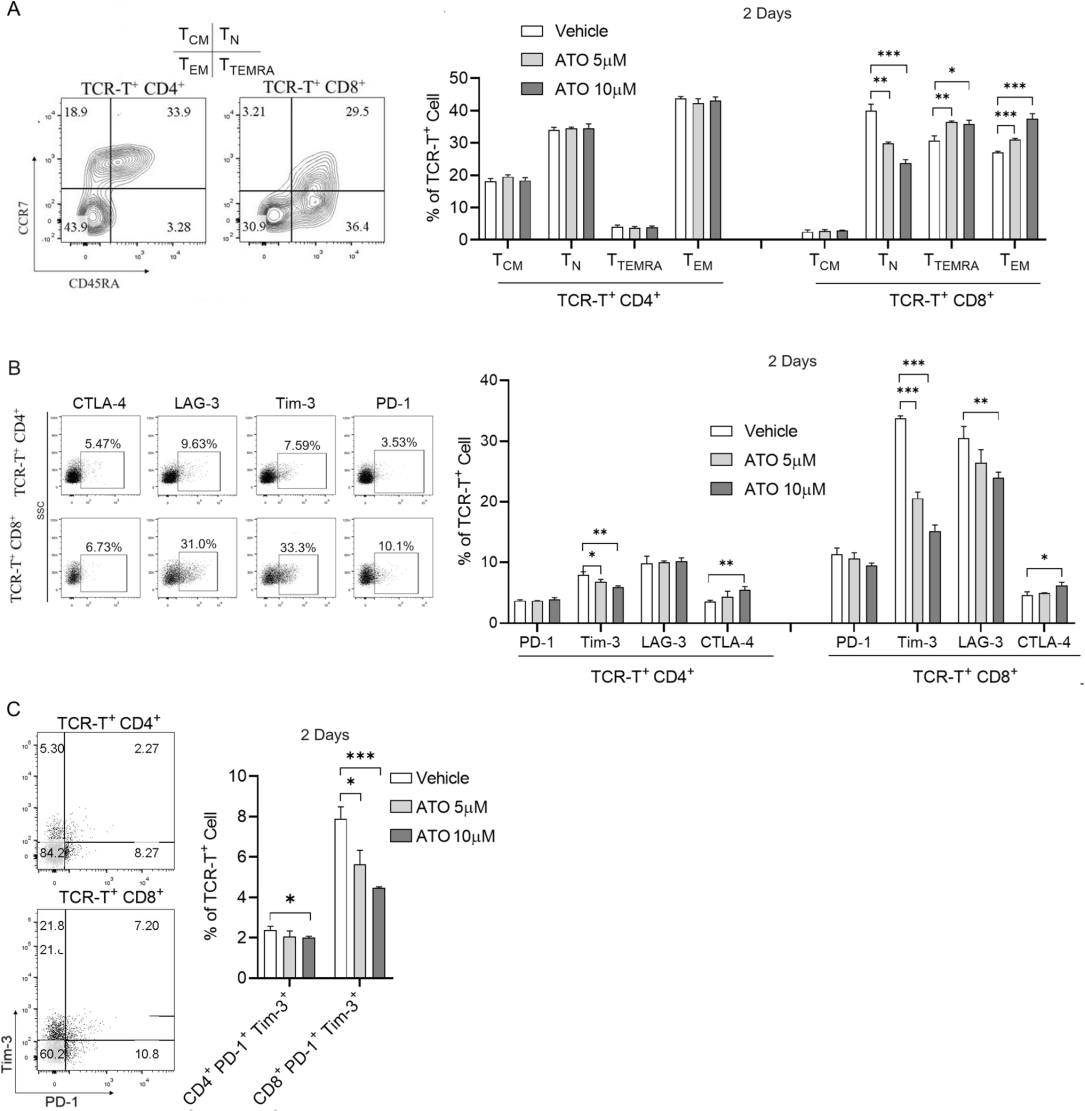
Effect of ATO on TCR-T subsets in response to cancer stimulation. **A** TCR-T cell memory subsets and **B** inhibitory receptor expression (PD-1, LAG-3, TIM-3 and CTLA-4) were analyzed by flow cytometry after TCR-T single stimulation with HepG2 cell line for 2 days in the presence of different concentrations of ATO. **C** Double positive PD-1^+^ TIM-3^+^ populations were analyzed on gated CD4^+^ or CD8^+^ TCR-T cells in (**B**). Each experiment was performed in triplicate. The experiment was repeated twice with similar data. Data are presented as mean ± SD. * for *p* < 0.05, ** for *p* < 0.01 and *** for *p* < 0.001

We further investigated the phenotypic changes of TCR-T cells in the presence of ATO during the process of tumor cell killing. The TCR-T cells were subjected to ATO for 9 days. No alterations in the differentiation of TCR T cells, either CD4^+^ or CD8^+^, were observed (Fig. S2A). Subsequently, TCR-T cells underwent single or repeated stimulation with HepG2 tumor cells in the presence of ATO, and we assessed the proportional changes among various T-cell subsets, including naïve T cells (T_N_; CD45RA^+^CCR7^+^), central memory T cells (T_CM_; CD45RA^−^CCR7^+^), effector memory T cells (T_EM_; CD45RA^−^CCR7^−^) and CD45RA^+^ effector memory cells (T_TEMRA_; CD45RA^+^CCR7^−^). As depicted in Fig. 2A, following a 2 days coculture with HepG2 (single stimulation), no significant difference was observed in the proportion of four subsets in CD4^+^ TCR-T cells with different concentrations of ATO. However, the proportions of CD8^+^ T_N_ were markedly reduced, but CD8^+^ T_EM_ and CD8^+^ T_TEMRA_ were increased in the ATO group compared to the vehicle group. These results indicated that ATO contributes more to the killing function of CD8^+^ TCR-T cells compared to CD4^+^ TCR-T cells, which may promote the differentiation of CD8^+^ T cells to generate a robust antigenic stimulus response. Furthermore, after 7 days (3 rounds) of repeated stimulation with HepG2, only CD4^+^ T_EM_ was increased in the ATO group (Fig. S2B), potentially indicating that ATO stimulates the differentiation of CD4^+^ TCR-T cells into more cytotoxic T cells.

Additionally, we evaluated the impact of ATO on the immune inhibitory receptors. The expression level of immune checkpoint molecules, including PD-1, TIM-3, LAG-3, and CTLA-4, was assessed by flow cytometry after single stimulation (Fig. 2B). In the case of CD4^+^ TCR-T cells, the expression of all four inhibitory receptors remained low (< 10%). Compared with the vehicle group, ATO treatment did not affect the expression of PD-1 and LAG-3 but led to decreased Tim-3 expression and upregulated CTLA-4 expression. Similarly, for CD8^+^ TCR-T cells, the expression of PD-1 and CTLA-4 was relatively low (< 13%) with ATO treatment resulting in an upregulation of CTLA-4 expression. Critically, TIM-3 and LAG-3 expression was significantly lower in the ATO-treated group (Fig. 2B). Because TIM-3^+^ T cells and the PD-1^+^ TIM-3^+^ double-positive cell population have diminished cytotoxicity and cytokine-producing capacity and thus may be attributed to the exhausted compartment [35]. Further analysis of PD-1^+^ TIM-3^+^ double-positive cells in CD8^+^ TCR-T cells revealed that ATO treatment significantly diminished the proportion of this subset of cells within CD8^+^ TCR-T cells (Fig. 2C). After 7 days of repeated antigen stimulation, CTLA-4 expression was downregulated by ATO treatment in CD4^+^ TCR-T cells, but PD-1 and Tim-3 CD4^+^ TCR-T cells were increased in the ATO-treated group compared to the vehicle group (Fig. S2C). This result aligns with the inference drawn from Fig. 2B, suggesting that ATO promotes the differentiation of CD4^+^ TCR-T cells into more cytotoxic T cells. As for CD8^+^ TCR-T cells, both PD-1 and CTLA-4 were reduced in the group with 10 μM ATO compared to the vehicle group (Fig. S2C). These findings indicate that ATO predominantly exerts a suppressive effect on inhibitory receptor expression when CD8^+^ TCR-T cells are stimulated with antigens.

### ATO combined treatment with TCR-T cells enhances suppression of HCC tumor growth in vivo

**Fig. 3:**
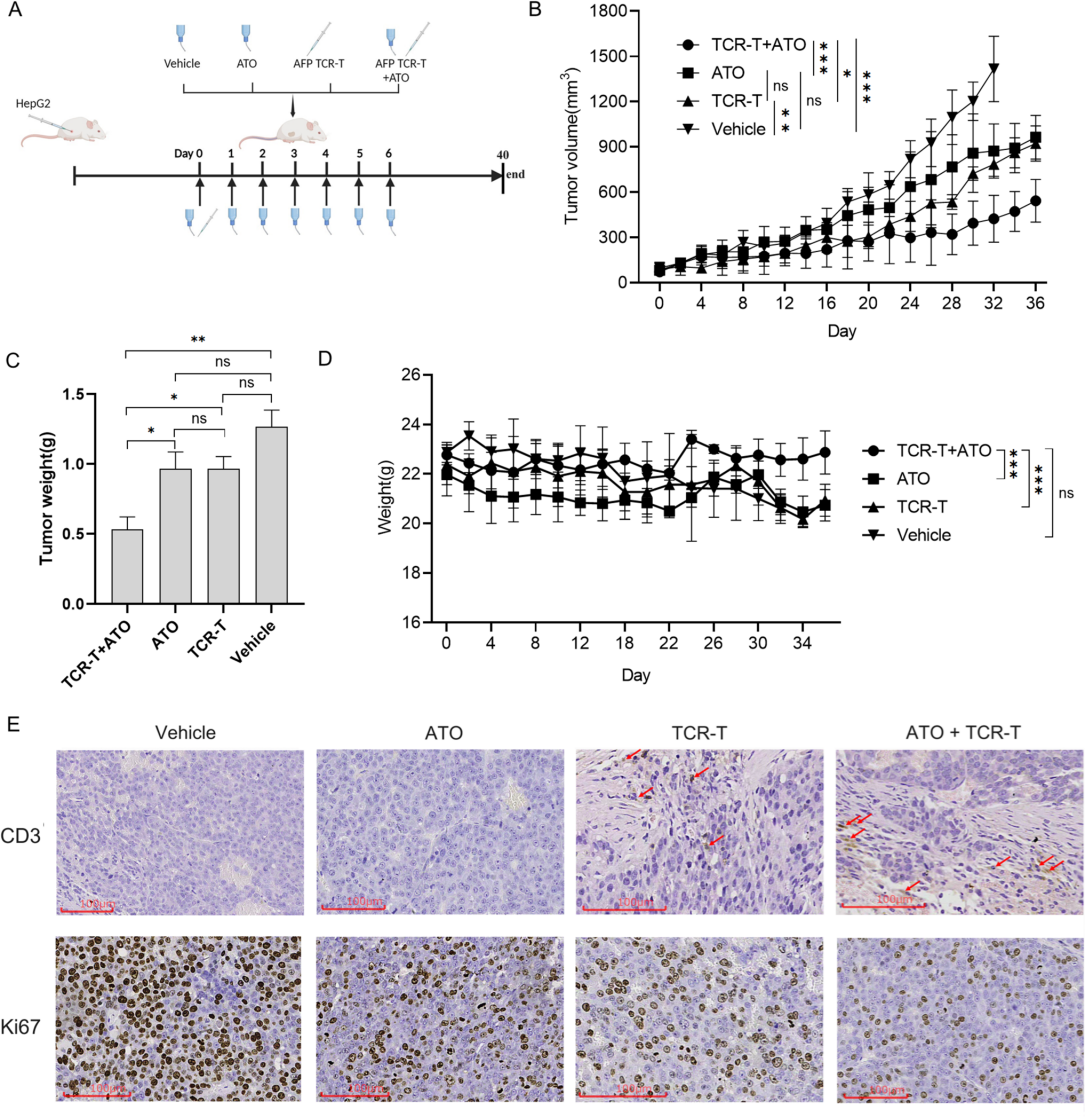
Direct comparison of the anti-HCC activity of ATO combined with AFP TCR-T therapy in HCC xenografts. **A** Protocol schema. B-NDG mice were engrafted with HepG2 cells (5 × 10^6^ cells/mouse, i.v.). Mice were randomized according to tumor burden, to receive vehicle, ATO 50 mg/Kg/day or AFP TCR-T cells 4 × 10^6^ CD8^+^ TCR-T cells/mouse. ATO and vehicle were continued for 7 days. **B** ATO combined with TCR-T cells treatment significantly inhibited tumor growth in vivo compared to the control (vehicle, ATO or TCR-T cells alone). Statistical significance was calculated by two-way ANOVA. **C** tumor weight on day 36 and **D** body weight change of mice bearing HepG2 xenografts after the treatment, and analyzed by two-way ANOVA analysis. Tumors from the TCR-T and ATO + TCR-T groups mice show infiltration of CD3^+^ T-cells (CD3, upper panel, indicated with arrow; scale bar: 100 μm) by IHC staining with anti-CD3 antibody. IHC staining with anti-Ki67 antibody showed that ATO + TCR-T treatment inhibited cell proliferation (Ki67, lower panel, scale bar: 100 μm). Data are presented as mean ± SD. * for *p* < 0.05, ** for *p* < 0.01 and *** for *p* < 0.001

To determine whether ATO can synergize effectively with TCR-T therapy in vivo, a subcutaneous xenograft tumor mouse model was successfully established with HepG2 cells. The schematic diagram of HepG2 model experimental procedures is illustrated in Fig. 3A. Compared to the control group (Vehicle, ATO or TCR-T cells treatment alone), ATO combined with TCR-T cells treatment synergistically inhibited tumor growth in the xenograft tumor mice (Fig. 3B). Consistently, tumor weight was higher in the control group than in the combination therapy group (Fig. 3C). Mice body weight of ATO combined with TCR-T cells treated tumor-bearing mice was significantly higher than those treated with either ATO or TCR-T cells alone, while maintaining a similar initial weight, implying that the combination of ATO and TCR-T cells has no obvious toxicity in experimental animals (Fig. 3D). To gain insight into the TCR-T cell infiltration of tumors, tumors were stained for human CD3. Figure 3E (upper panel) shows representative images of a positively stained tumor from mice in the TCR-T group and ATO + TCR-T groups, while this staining was absent in the vehicle and ATO groups. Moreover, ATO + TCR-T also demonstrated an inhibitory effect on cell proliferation in vivo, as corroborated by the assessment of Ki67 expression (cell proliferation marker) (Fig. 3E, lower panel).

### ATO combined with TCR-T cells activated ferroptosis-related pathway

**Fig. 4:**
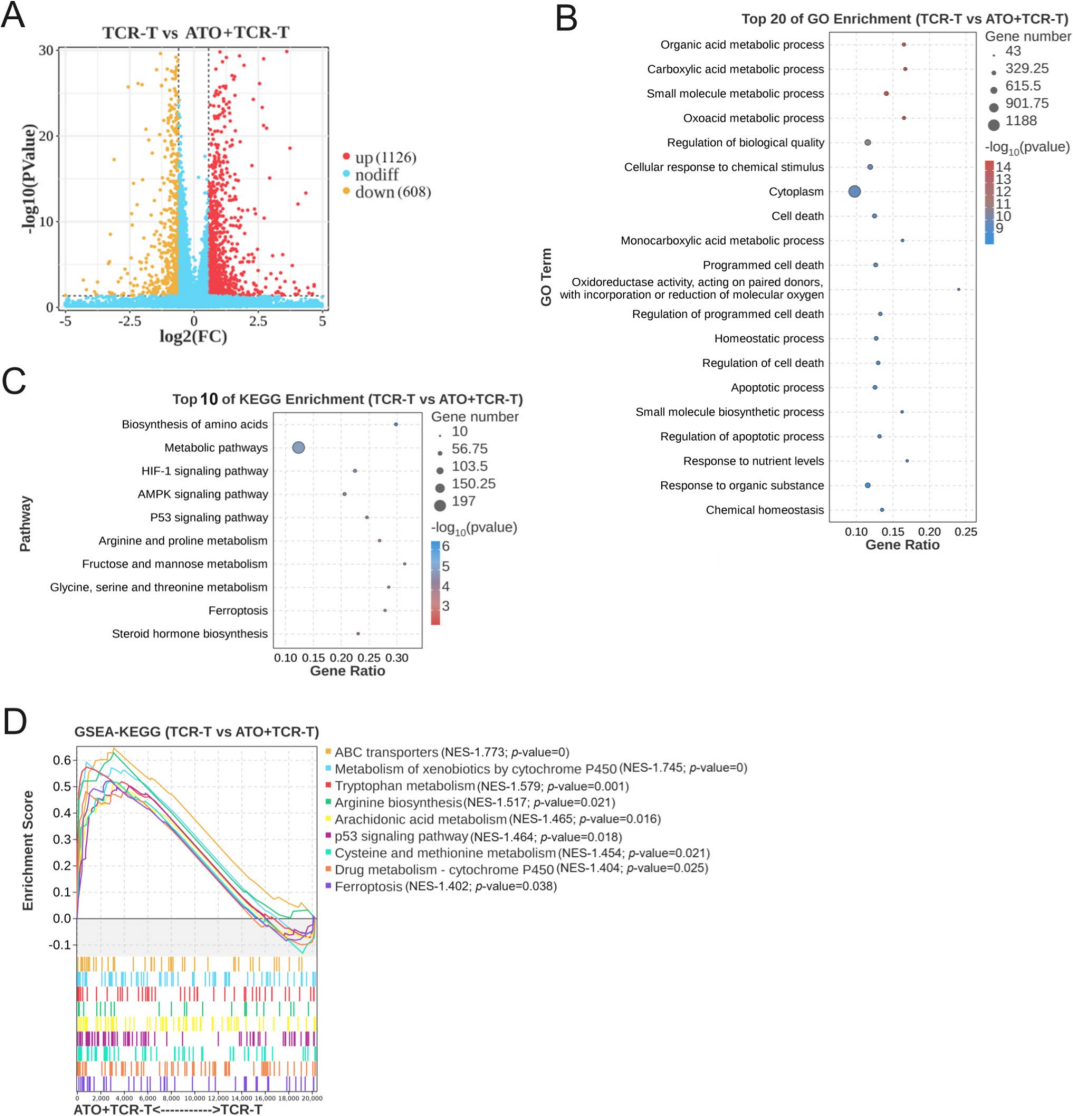
Transcriptomic analysis of the effects of ATO combined with AFP TCR-T. HepG2 cells were treated with 10 μM ATO, 1 × 10^6^ TCR-T cells (E∶T = 1∶1) or their combination. Transcriptome sequencing experiments were performed to detect mRNA expression levels (*n* = 3). **A** DEGs (|log2FC|> 0.58, *p* < 0.05) containing upregulated genes (red) and downregulated genes (yellow) were visualized by volcano plot based on gene expression difference (log2fold change, x axis) and statistical significance (− log10 *p* value, *y* axis). **B** GO analysis and **C** KEGG pathway enrichment analysis of DEGs. **D** GSEA of the KEGG signaling pathway activated in the ATO + TCR-T group compared to TCR-T group

To elucidate the underlying mechanism of ATO enhancement TCR-T cells anti-tumor efficacy, we conducted RNA-sequencing (RNA-seq) on HepG2 cells treated separately with 10 μM ATO, TCR-T cells, and their combination for 6 h. A total of 20 284 genes of tumor cells were analyzed and significant down/up-regulating genes were prominently clustered in the ATO + TCR-T group vs TCR-T group and ATO group vs vehicle group. The differentially expressed genes (DEGs) (|log2FC|> 0.58, *p* < 0.05) among the groups were visualized in the volcano plots (Figs. 4A, S3A). Compared to the vehicle group, 1 388 genes were up-regulated and 1 128 genes were down-regulated in the ATO group (Fig. S3A). In the ATO + TCR-T combined treatment group, 1 126 genes were upregulated and 608 genes were downregulated compared to the TCR-T monotherapy group (Fig. 4A). Subsequently, we performed GO analysis and KEGG pathway enrichment analysis based on the DEGs (Figs. 4B, C, S3B, C). The GO analysis revealed that the DEGs in the vehicle vs ATO and TCR-T vs ATO + TCR-T were both involved in some cell death biological processes, such as “Programmed cell death” and “Cell death” (Figs. 4B, S3B). Interestingly, KEGG pathway enrichment analysis further showed that both comparisons were implicated in the ferroptosis signaling pathway (Figs. 4C, S3C). The gene set enrichment analysis (GSEA) also discovered that the differentially expressed molecules were enriched in the arachidonic acid metabolism, p53 signaling pathways, cysteine and methionine metabolism, ferroptosis and other pathways closely associated with ferroptosis (Fig. 4D). Taken together, these results suggested that ferroptosis emerged as the most significantly altered cell death form for ATO + TCR-T treatment of HCC.

### ATO combined treatment with TCR-T induced ferroptosis in HCC

**Fig. 5:**
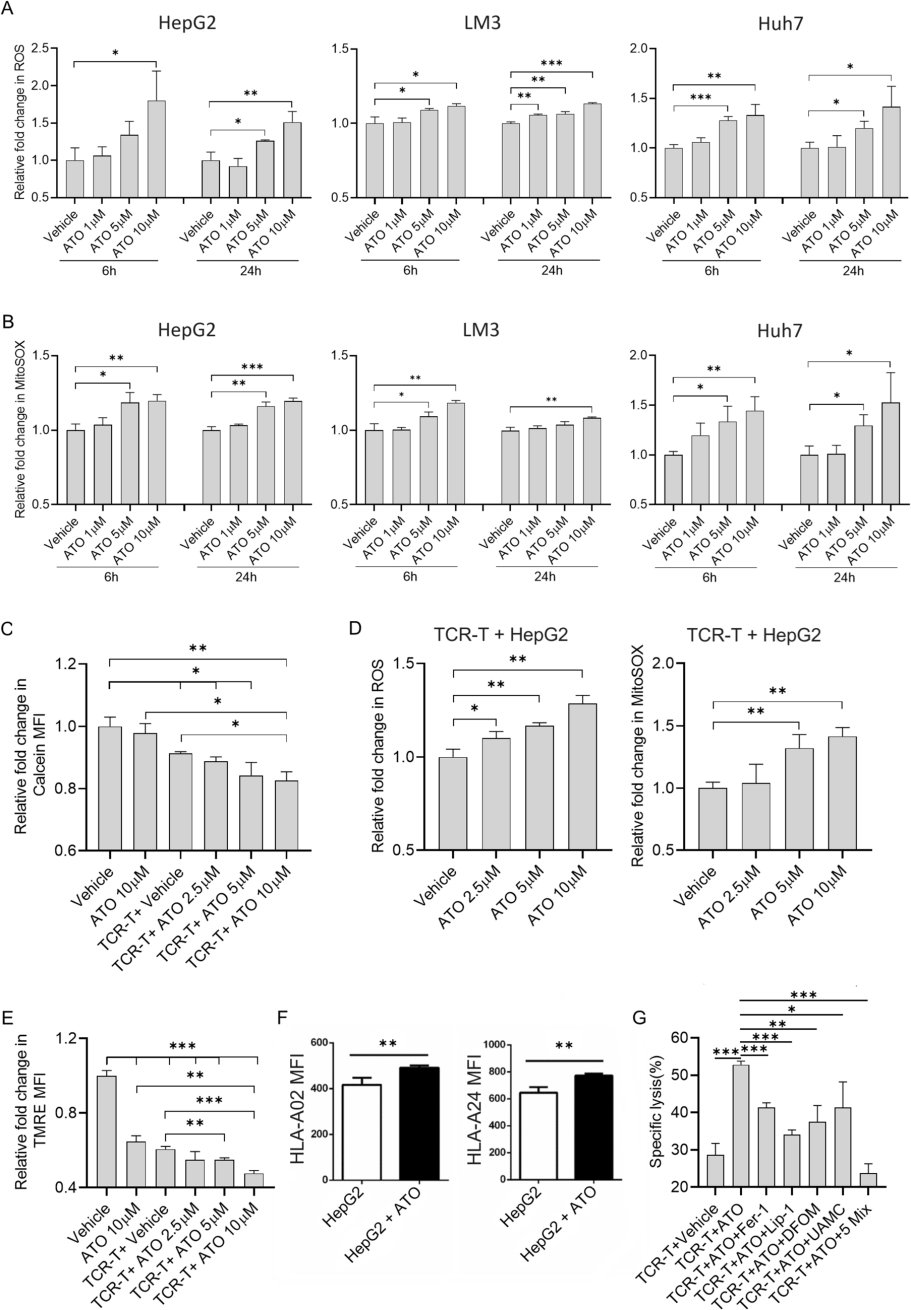
ATO combined treatment with TCR-T enhanced ferroptosis of HCC through excessive ROS accumulation. **A**–**F** Mean intensities of fluorescence observed by flow cytometry. **A**, **B**, **D** ROS assay and MitoSOX assay to evaluate intracellular ROS and mitochondrial ROS levels of HepG2, LM3, and Huh7 cells after treated with different concentrations of ATO alone for 6 and 24 h (**A**, **B**) or combined with TCR-T cells for 6 h (**D**). **C** Calcein-AM assay to evaluate the LIP levels of HepG2 cells after ATO and TCR-T cells treatment for 12 h by flow cytometry. **E** TMRE assay to evaluate the MMP levels of HepG2 cells after ATO and TCR-T cells treatment for 12 h by flow cytometry. **F** Class I molecule expression was detected by flow cytometry after HepG2 treatment with 10 μM ATO. **G** HepG2 cells were treated with TCR-T cells (E∶T = 0.5∶1) in the presence of ATO with or without the indicated inhibitors for 24 h and cell Lysis was assayed by LDH. ATO = 10 μM; Fer-1 (Ferrostatin-1) = 2 μM; Lip-1(Liproxstatin-1) = 2 μM; DFOM (Deferoxamine mesylate) = 10 μM; UAMC (UAMC-3203) = 1 μM; 5 Mix = ATO + Fer-1 + Lip-1 + DFOM + UAMC. Each experiment was performed in triplicate. The experiment was repeated twice with similar data. Data are presented as mean ± SD. * for *p* < 0.05, ** for *p* < 0.01 and *** for *p* < 0.001

We suspect that ATO may promote TCR-T anti-tumor function by rendering tumor cells more sensitive to ferroptosis. Firstly, ATO has been shown to increase ROS production in cancer cells in preclinical studies [24, 36, 37]. We also found ATO dose-dependently increased intracellular (Figs. 5A, S4A, C) and mitochondrial (Figs. 5B, S4A, D) ROS levels in HepG2 cells, HCC-LM3, and Huh7 cells since 6 h. It is essential to note that ROS accumulation is a primary trigger for ferroptosis [38]. Secondly, Wang et al. have identified that activated CD8^+^ T cells could induce tumor cell ferroptosis by generating IFN-*γ* [30]. Given that we also found ATO-enhanced IFN-*γ* production and secretion by TCR-T cells (Fig. 1D, E), we hypothesized that ATO could enhance the induction of ferroptosis by TCR-T cells via increased ROS accumulation. To test this hypothesis, we initially measured the content of LIP in HepG2 after co-treatment with ATO and TCR-T cells for 12 h by Calcein-AM [39]. The results showed that ATO could diminish HepG2 Calcein-AM fluorescence in a dose-dependent manner, indicating increasing intracellular levels of LIP in HepG2 (Figs. 5C, S4E). Then, an increased accumulation of intracellular and mitochondrial (Figs. 5D, S4B, F) ROS was observed in cells co-treated with ATO and TCR-T cells compared to ATO-treated cells. Next, we assessed ΔΨm levels after co-treatment with ATO and TCR-T cells for HepG2 (Figs. 5E, S4G). The result showed that the decrease in ΔΨm was ATO dose-dependent. Moreover, we also found that ATO treatment increased cell surface HLA-I expression in HepG2 (Fig. 5F). This may enhance TCR-T recognition and promote the production of IFN-*γ*. Given that mitochondrial ROS and IFN-*γ* have been reported to trigger ferroptosis [29, 30], we explored whether ferroptosis inhibitors could rescue cell death induced by ATO in combination with TCR-T cells. All four inhibitors inhibited ATO-enhanced killing to varying degrees, and in particular, the ATO-promoting effect was completely inhibited when the four inhibitors were treated together in the HepG2 cells (Fig. 5G). These findings confirm that TCR-T cell-derived IFN-*γ* in combination with ATO-induced ROS accumulation makes tumor cells more sensitive to ferroptosis.

## Discussion

**Fig. 6:**
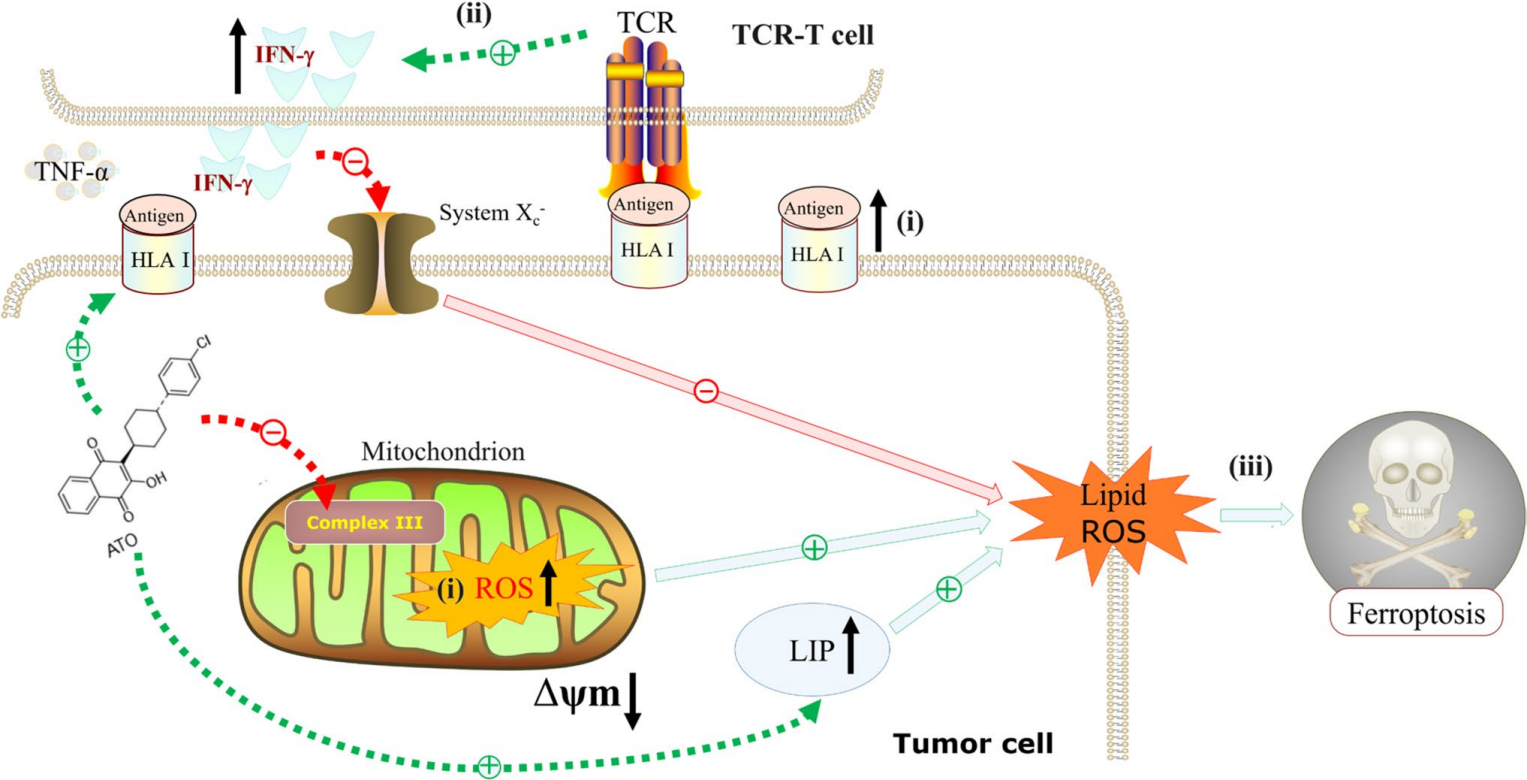
A schematic representation of atovaquone improves TCR-T against HCC by exacerbating tumor excessive ROS-induced ferroptosis. We propose a sequence of events in which: (i) ATO induces tumor cells ROS accumulation and upregulates HLA-I expression, that (ii) promotes TCR-T recognition and IFN-*γ* release, leading to (iii) triggering and enhancement of IFN-*γ*-mediated ferroptotic cell death. ATO, atovaquone; ROS, reactive oxygen species; Δψm, mitochondrial transmembrane potential; LIP, labile iron pool

Ferroptosis is a novel form of regulated cell death, it is a promising approach to T cell-based anti-tumor immunotherapeutic for novel observation suggested that T cell initiated the tumor cell ferroptosis [40]. As ACTs have become the focus of novel cancer therapies [15], the combination of ACTs with ferroptosis-targeting drugs presents a theoretically plausible approach to enhance anti-tumor immunotherapy [40]. In the present study, we discover that TCR-T cells-produced IFN-*γ* in combination with ATO-induced ROS accumulation directly boosts ferroptosis in HCC. This mechanism underpins the TCR-T cell-mediated eradication of tumors in the presence of ATO (Fig. 6).

CD8^+^ T cells play a central role in antitumor immunity, but their activity is limited in the tumor microenvironment (TME) [41]. TCR-T therapies are generally composed of CD4^+^ TCR-T cells and CD8^+^ TCR-T cells, both of which have effective antitumor cytotoxic activities in vitro and in vivo [42]. Both our results (Fig. 1E) and those of Liang et al. [42] suggests that the CD4^+^ TCR-T, like the CD8^+^ TCR-T, also produces IFN-*γ*. A recent study shows that tumor cell-intrinsic IFN-*γ* signaling was necessary for optimal T cell-based-mediated anti-tumor cell [43]. Moreover, Wang et al. showed that activated CD8^+^ T cells could induce tumor cell ferroptosis through the generated IFN-*γ* targeting the X_C_^−^ system [30]. While IFN-γ alone cannot directly trigger ferroptosis, its combination of ferroptosis inducers, such as synthetic-molecule or cellular metabolite (e.g., arachidonic acid) directly triggers tumor cell ferroptosis [30, 44]. In addition, since IFN-*γ* is often produced by activated CD8^+^ T cells, paucity of T cell infiltration, insufficient IFN-*γ* generation, loss of IFN-*γ* gene signaling, or ferroptosis inducer deficiency can result in bypassing the aforementioned immune-related tumor ferroptotic mechanism, allowing tumor progression. Hence, our data suggest that tumor ferroptosis deficiency may be a previously unrecognized feature of ineffective T-cell-based immunotherapies. In this study, we discovered that ATO increased HLA-I expression in cancer cells (Fig. 5F) and stimulated IFN-*γ* secretion in TCR-T cells when co-cultured with cancer cells (Fig. 1D). This is crucial for facilitating the triggering of IFN-*γ*-mediated ferroptosis.

It is well known that CTLs kill targets via the perforin-granzyme-caspase-mediated apoptosis [45]. Nevertheless, direct evidence of the connection between ferroptosis and anti-tumor immunity was not available until Wang et al. reported that CD8^+^ T cells induce ferroptosis in tumor cells [30]. A growing body of evidence points toward ferroptosis as an emerging category of cell death within the spectrum of immunogenic cell death (ICD). Notably, ferroptotic demise of tumor cells has been identified as a significant source of danger-associated molecular patterns (DAMPs), encompassing molecules such as HMGB1, DNA, ATP, as well as lipid oxidation products like LTB4 and PGE2, particularly in the context of cancer therapy [46]. Moreover, multiple of evidence suggest that ferroptosis can limit the function of immunosuppressive cells, and increase immune cell infiltration, thereby potentially surmounting primary resistance to immunotherapy and bolstering the efficacy of ICIs [47]. In light of the established understanding of ferroptosis and its implications for antitumor immunity, we posit that TCR-T cells function as a trigger for ferroptosis in tumor cells, thereby resulting in ferroptotic tumor cell death and subsequent tumor antigen release and turning “cold” tumors into “hot” ones by reshaping the tumor microenvironment. In support of this notion, we demonstrate that the combination of ATO and TCR-T therapy enhances CTL-mediated tumor cell death via induction of ferroptosis. It is noteworthy, however, that our investigation utilized immunodeficient mice, necessitating future exploration of the therapeutic potential of ferroptosis induction within an immunocompetent murine model.

The burgeoning elucidation of ferroptosis mechanisms has catalyzed the repositioning of approved drugs to stimulate this form of regulated cell death, either in combination with conventional anticancer agents or as a foundation for therapeutic innovations [48]. Among these, ATO, an established anti-malaria drug that was authorized by FDA in 1995 [16], has already been repurposed for its novel anti-tumor effects in various cancer cells [18–20, 37]. Research has substantiated ATO's antineoplastic properties, attributed to its capacity to induce DNA damage [21] and surmount chemoresistance in HCC [49]. Studies have shown that ATO can also inhibit cancer cell viability by targeting the mitochondrial complex III [19, 22], which increases the ROS levels within cancer cells. In addition, it has been reported that ATO can increase oxidative stress in vitro [24] and reduce hypoxia in vivo [19], which may enhance T cell-based cancer immunotherapy by alleviating the hypoxic conditions within the tumor microenvironment [50]. As we know, ferroptosis is a ROS-dependent form of cell death, ROS-inducing drugs (such as doxorubicin, gemcitabine, paclitaxel, 5-fluorouracil, bortezomib, and arsenic trioxide) in combination with Cyst(e)inase is considered to be new therapeutic opportunities for ferroptosis-based anticancer therapy [51]. Therefore, given its capability to induce ROS, ATO represents a safe and efficient drug candidate for targeting ferroptosis by promoting the excessive production and accumulation of ROS. Our research findings strongly propose that the combined application of ATO and TCR-T therapy is instrumental in instigating ferroptosis within tumor cells, ultimately leading to a remarkable enhancement in the suppression of HCC, both in controlled in vitro experiments and preclinical in vivo settings.

### Supplementary Information

Below is the link to the electronic supplementary material.

Supplementary file 1 (PDF 860 kb)

## Data Availability

The datasets generated during and/or analyzed during the current study are available from the corresponding author on reasonable request.

## Declarations

### Supplementary information

Cancer Immunology, Immunotherapy (submitted in 2023) – Anan Chen et al.
