# Supplementary material for: Atovaquone enhances antitumor efficacy of TCR-T therapy by augmentation of ROS-induced ferroptosis in hepatocellular carcinoma
Source: Cancer Immunol Immunother. 2024 Feb 13;73(3):49. doi: 10.1007/s00262-024-03628-2 (PMC10864481; doi:10.1007/s00262-024-03628-2)
Supplement: Supplementary file 1 — (PDF 860 kb) [file 262_2024_3628_MOESM1_ESM.pdf]

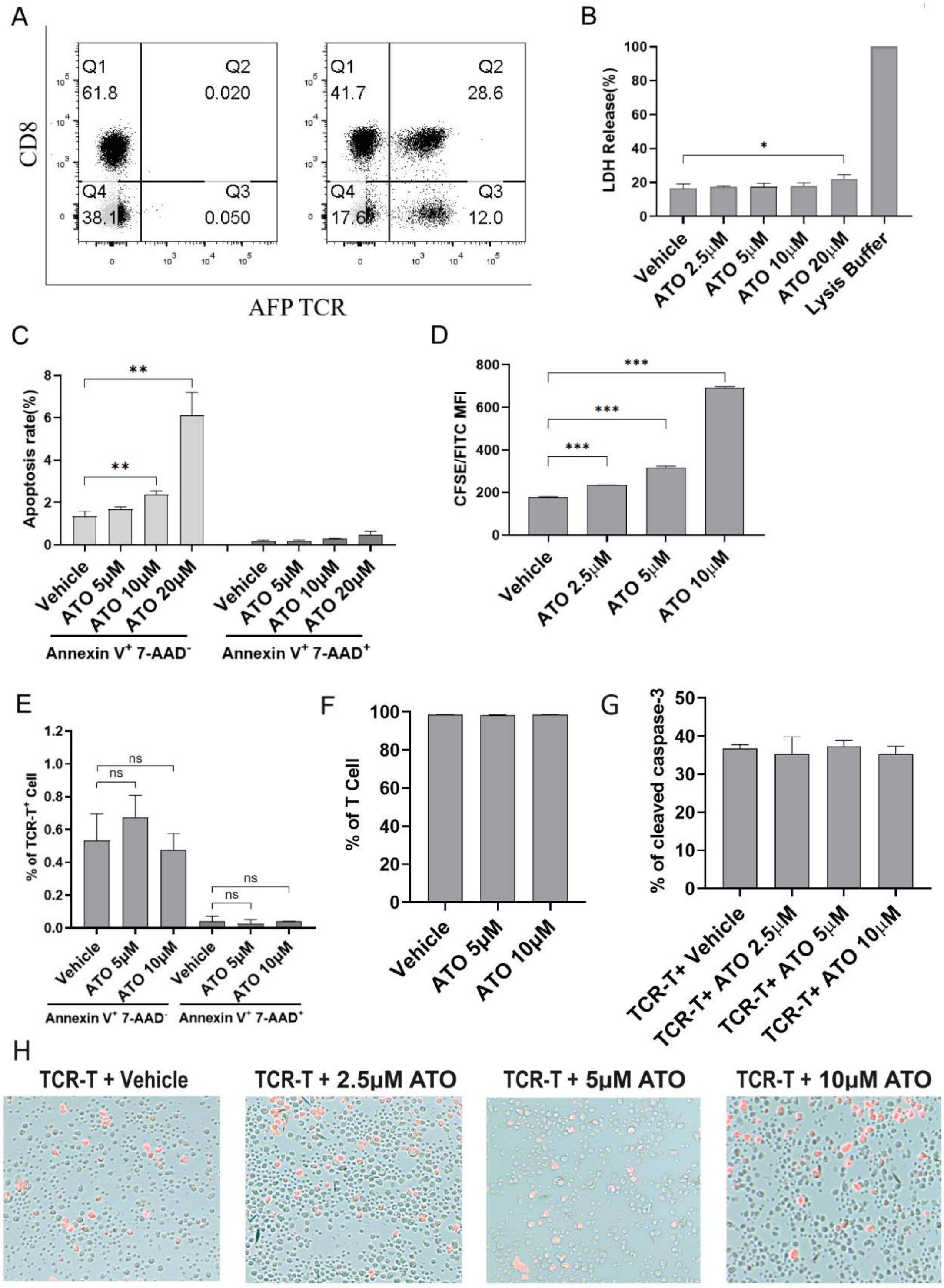

Fig. S1 Effect of ATO in TCR-T cell function in vitro. (A)Staining of AFP TCR-T cells or nontransduced T cells with anti-mouse TCRβ-chain antibody and CD8 antibody. (B) HepG2 cells were incubated with different doses of ATO or vehicle 24h at 37°C. DMSO was used as a vehicle. The cells incubated with a 10%Triton X-100 solution was used as a positive control of cytotoxicity. (C)ATO induced HepG2 cells apoptosis in a concentration-dependent manner for 48h. (D) Inhibition of HepG2 cell proliferation was examined after 4-day culture with ATO. (E) ATO

could not induced TCR-T cells apoptosis for 48h. (F) After ATO or vehicle treated TCR-T cells for 9 days, Cell viability was detected by flow cytometry with 7-AAD staining. (G)Cleaved caspase-3 expression was detected by flow cytometry after HepG2 treated with TCR-T or combined with different concentrations of ATO for 6h (E：T=1：1). (H) Representative fluorescence microscopy images (10×) of (G). Each experiment was performed in triplicate. The experiment was repeated twice with similar data. Data are presented as mean ± SD. $p < 0.05$, ** for $p < 0.01$ and *** for $p < 0.001$.

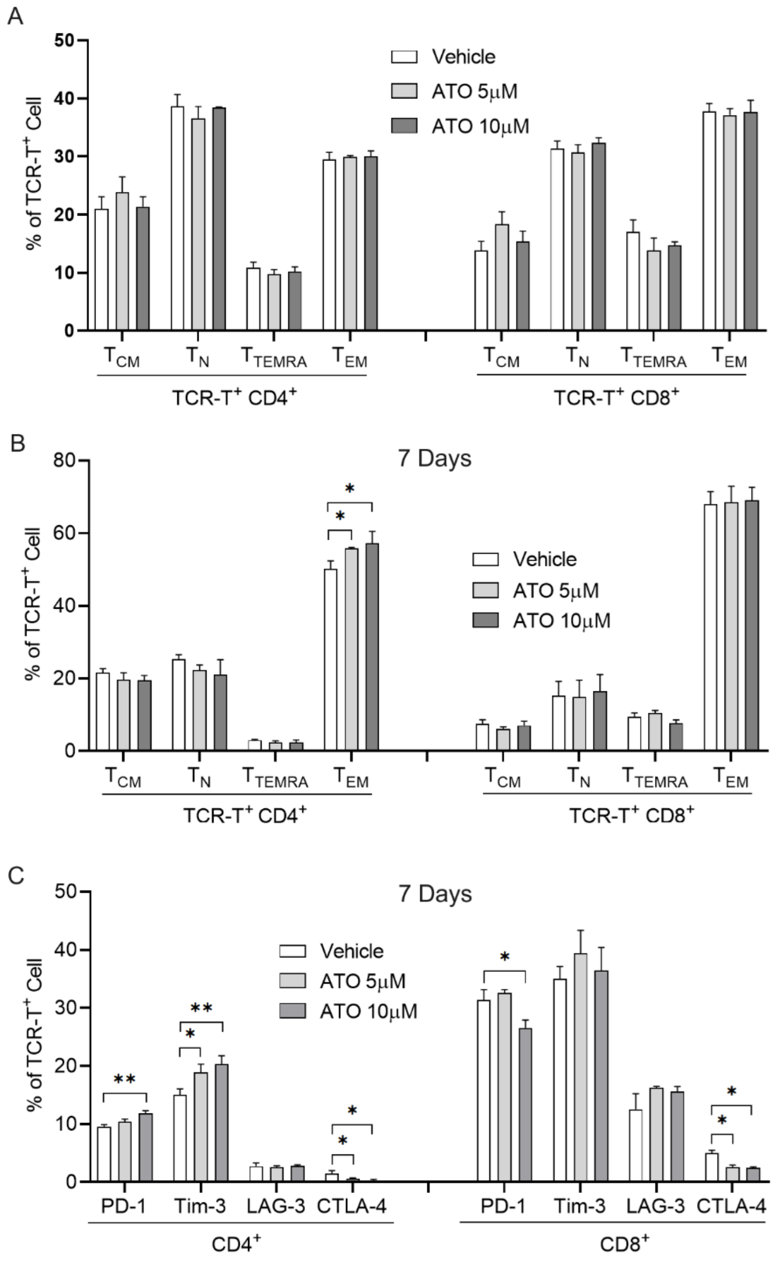

Fig. S2 Effect of ATO on TCR-T subsets in response to cancer stimulation. (A)TCR-T cells memory subsets were analyzed by flow cytometry after treated with ATO for 9 days. Medium was changed every two days and maintain the appropriate ATO concentration. (B) TCR-T cell memory subsets and (C) inhibitory receptor expression (PD-1, LAG-3, TIM-3 and CTLA-4) were analyzed by flow cytometry after TCR-T single stimulation with HepG2 cell line for 3 rounds of repeated stimulation over 7 days in the presence of different concentrations of ATO. Each experiment was performed in triplicate. The experiment was repeated twice with similar data, Data are presented as mean ± SD. $p < 0.05$, ** for $p < 0.01$ and *** for $p < 0.001$.

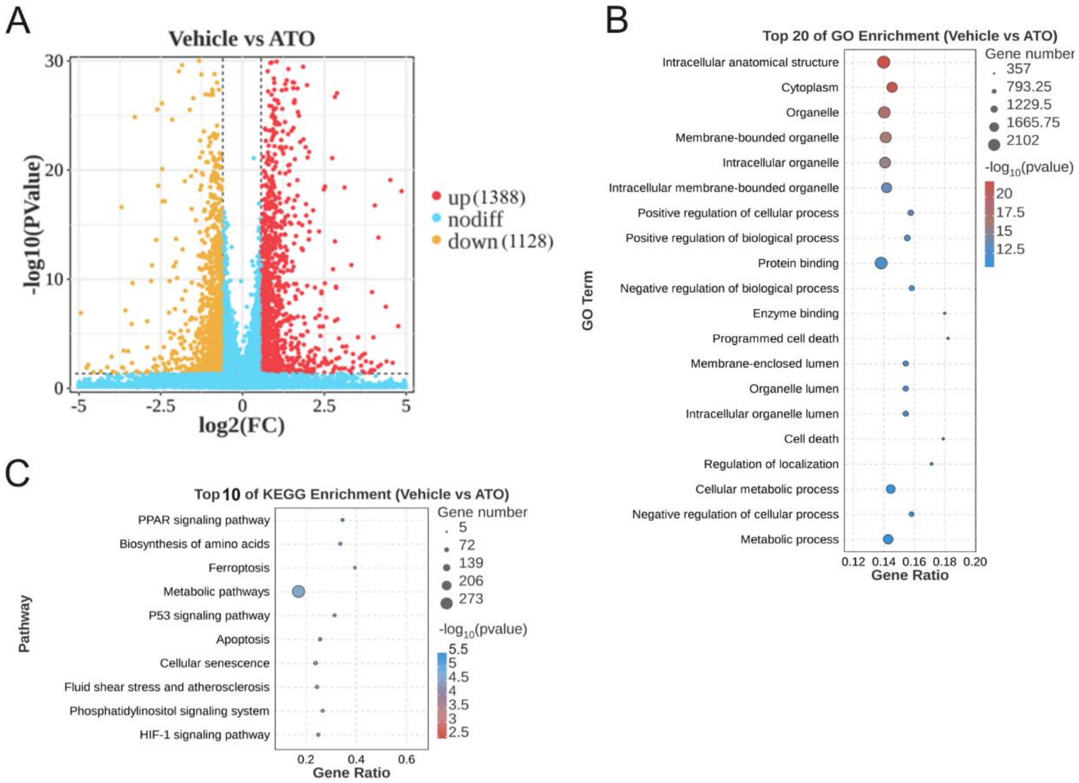

Fig. S3 Transcriptomic analysis of the effects of ATO combined with AFP TCR-T. (A) DEGs (|log2FC|>0.58, $p <$ 0.05) containing upregulated genes (red) and downregulated genes (yellow) were visualized by volcano plot based on gene expression difference (log2fold change, x axis) and statistical significance (-log10 pvalue, y axis). (B) GO analysis and (C) KEGG pathway enrichment analysis of DEGs.

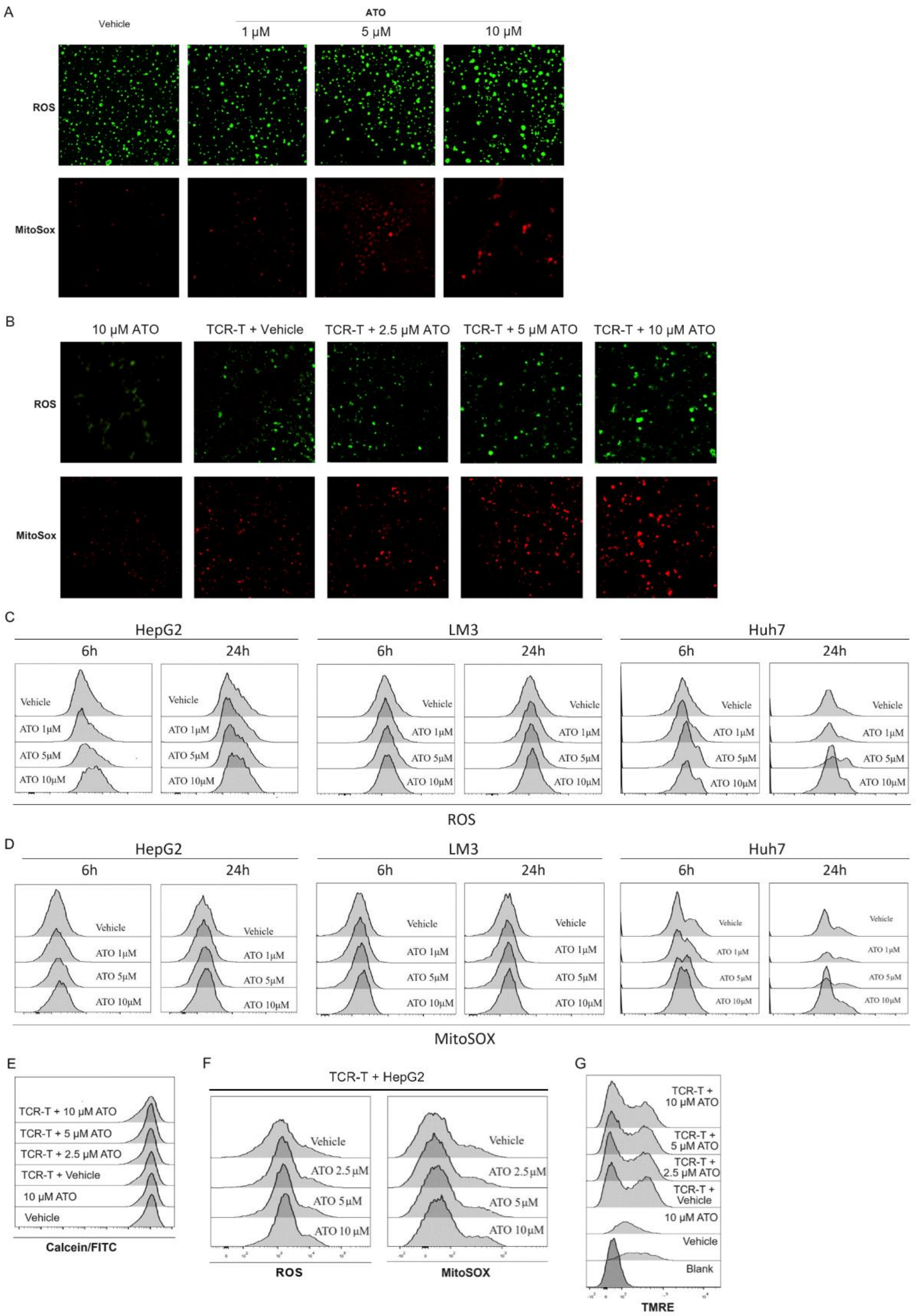

Fig. S4 ATO combined treatment with TCR-T enhanced ferroptosis of HCC through excessive ROS accumulation. (A) and (B) Representative fluorescence microscopy images (20×) of HepG2 stained with fluorescent dyes. Green color indicates HepG2 cells stained with DCFH-DA for intracellular ROS and red color indicates HepG2 cells stained with MitoSOX red indicator for mitochondrial ROS. Effect of different concentrations of ATO alone (A) or combined with TCR-T cells (E：T=1：2) (B) on HepG2 cells after 6h treatment. (C-G) Representative flow cytometry histograms of Fig. 5A-E.
